# β‐Hydroxybutyrate Acts as an Exercise Mimetic to Protect the Aging Liver

**DOI:** 10.1111/acel.70314

**Published:** 2025-12-08

**Authors:** Ke Li, Lian Wang, Danlin Zhu, Wenhong Wang, Yifan Guo, Haoyang Gao, Muge Zhou, Weihua Xiao

**Affiliations:** ^1^ Shanghai Key Lab of Human Performance (Shanghai University of Sport) Shanghai University of Sport Shanghai China; ^2^ The Key Lab of Exercise and Health Sciences of Ministry of Education Shanghai University of Sport Shanghai China; ^3^ Department of Neurology, Medical College of Georgia Augusta University Augusta Georgia USA; ^4^ School of Elderly Care Services and Management Nanjing University of Chinese Medicine Nanjing China; ^5^ Rehabilitation Center Children's Hospital of Fudan University Shanghai China

**Keywords:** β‐hydroxybutyrate, aerobic exercise, cGAS‐STING, inflammation, lipid deposition, liver aging, PANoptosis

## Abstract

Liver aging is characterized by pathological features including lipid deposition, exacerbated chronic inflammation, and increased cell death. Although exercise intervention has been proven effective in delaying liver aging, its fundamental biochemical mechanism remains unclear. This study utilized a naturally aged mouse model and an in vitro cellular senescence system to reveal, for the first time, the cascade mechanism by which β‐hydroxybutyrate (β‐HB), a core protective mediator induced by aerobic exercise, delays liver aging through regulating the macrophage–hepatocyte crosstalk. Within the aging microenvironment, disturbance of mitochondrial homeostasis results in the cytosolic release of mtDNA, which activates the cGAS‐STING signaling pathway and drives macrophage polarization towards the pro‐inflammatory M1 phenotype. M1 macrophages subsequently indirectly induce hepatocyte lipid metabolic dysregulation and initiate PANoptosis. Aerobic exercise stimulates the production of endogenous β‐HB, which protects mitochondrial function, inhibits the activation of the cGAS‐STING pathway in macrophages, facilitates macrophages transformation into the anti‐inflammatory M2 phenotype, and ultimately indirectly ameliorates hepatocyte lipid deposition and PANoptosis. Additionally, exogenous β‐HB administration efficiently mimics the endogenous ketogenic effect of aerobic exercise, restoring mitochondrial homeostasis, mitigating inflammation, and reducing PANoptosis levels in the liver of aged mice. This study elucidates the molecular mechanisms by which exercise‐induced endogenous β‐HB confers hepatoprotection. We establish β‐HB as an exercise mimetic, exerting its protective effects on the aging liver through targeted inhibition of the innate immune hub STING. These findings provide a robust theoretical and experimental foundation for the translational application of β‐HB in clinical nutritional strategies for aging intervention.

## Graphical abstract

β‐hydroxybutyrate (β‐HB) acts as an exercise mimetic to protect the aging liver. In aged models, exercise‐induced β‐HB delays liver aging by reversing macrophage senescence, blocking mtDNA/cGAS‐STING‐driven inflammation, and alleviating hepatocyte lipid deposition/PANoptosis.

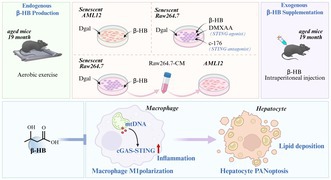

**Abbreviations**
β‐HB: β‐hydroxybutyrate
AIM: absent in melanoma
ALT: alanine aminotransferase
ALD: alcoholic liver disease
AST: aspartate aminotransferase
CM: conditioned medium
DAPM: damage‐associated molecular pattern
HCC: hepatocellular carcinoma
HDL‐C: high‐density lipoprotein cholesterol
LDL‐C: low‐density lipoprotein cholesterol
MASLD: metabolic dysfunction‐associated steatotic liver disease
mtDNA: mitochondrial DNA
NLRP12: NLR family pyrin domain containing 12
SA‐β‐Gal: senescence β‐galactosidase
TC: total cholesterol
TGF‐β: transforming growth factor‐bet
TG: triglyceride
ZBP1: Z‐DNA binding protein 1

## 1 Introduction

Aging causes substantial alterations in the liver, increasing its vulnerability to damage and worsening the prognosis of conditions such as metabolic dysfunction‐associated steatotic liver disease (MASLD), alcoholic liver disease (ALD), and hepatocellular carcinoma (HCC) (Chen and Wang 2025; Ren et al. 2022; He, Su, et al. 2023). Key characteristics of liver aging include metabolic disturbances, mitochondrial dysfunction, and chronic inflammation, which together constitute the early phenotypes of various liver diseases (Wang et al. 2024). Lipotoxicity and mitochondrial dysfunction interact and collectively drive age‐associated inflammatory responses. Recent studies indicate that the senescent liver secretes transforming growth factor‐beta (TGF‐β) into the circulatory system, hence inducing senescence and functional impairment in other organs, including the kidneys and brain (Kiourtis et al. 2024). Consequently, liver aging significantly contributes to the induction of systemic aging.

The cGAS‐STING pathway functions as a critical immune sensor for aberrant cytosolic DNA. Mitochondrial dysfunction during aging leads to the release of mitochondrial DNA (mtDNA), activating the cGAS‐STING pathway and thereby promoting chronic inflammation (Jiménez‐Loygorri et al. 2024). cGAS‐STING has been established as a key mechanism driving senescence in organs including the brain, retina, and kidneys (Jiménez‐Loygorri et al. 2024; Gulen et al. 2023). However, the alterations and functional significance of the cGAS‐STING pathway in the aging liver remain unclear. Recent research indicates that cGAS‐STING expression is elevated in the liver tissues of patients with MASLD. Moreover, the activation of STING in liver macrophages mediates hepatic inflammation and fibrosis in mouse models (Luo et al. 2018). PANoptosis is a recently identified inflammatory form of programmed cell death, characterized by the simultaneous or successive activation and integration of pyroptosis, apoptosis, and necroptosis, resulting in a robust inflammatory response (Sun et al. 2024). Recent research indicates that PANoptosis is crucial in inflammatory and metabolic liver diseases and offers considerable promise for liver disease therapy (Xiong et al. 2025). Nonetheless, it is uncertain if the PANoptosis pathway is engaged in the aging liver and whether its activation is contingent upon macrophage cGAS‐STING signaling. Consequently, clarifying the causes of liver aging and developing appropriate therapies is of paramount importance for delaying hepatic senescence and promoting healthy aging.

Dietary and lifestyle interventions represent crucial strategies for delaying liver aging. Numerous preclinical and clinical investigations suggest that aerobic exercise is associated with a reduction in cellular senescence markers, although the precise mechanisms underlying its specific effects remain incompletely elucidated (Zhang et al. 2022). The ketone body β‐hydroxybutyrate (β‐HB), once regarded merely as an alternative energy source generated during lipid metabolism in exercise, has recently gained significant attention as a signaling molecule with potential anti‐aging properties (Wang et al. 2021). β‐HB demonstrates protective effects in various liver disease models and is markedly upregulated in strategies known to delay liver aging, including physical exercise, fasting, and caloric restriction (Li et al. 2023). Notably, research indicates that β‐HB suppresses the cGAS‐STING pathway in models of cisplatin‐induced acute kidney injury, reducing inflammation and apoptosis (Luo et al. 2022). This indicates that β‐HB may be a crucial mediating molecule implicated in the anti‐liver aging effects elicited by exercise.

Our study employs a naturally aged mouse model to clarify the mechanism by which aerobic exercise enhances liver protection via increased β‐HB levels. We induced senescence in AML12 hepatocytes and Raw264.7 macrophages at the cellular level to examine the role of β‐HB. Our research demonstrated that β‐HB can exert anti‐inflammatory effects by decreasing mtDNA release in senescent macrophages and inhibiting the cGAS‐STING signaling pathway, which subsequently leads to a reduction in lipid deposition and PANoptosis in hepatocytes. Finally, by exogenously supplementing β‐HB to mimic exercise‐induced ketogenesis, we validated the protective effects of β‐HB on the liver in aged mice. By bridging human transcriptomic data with mechanistic models, we elucidate the molecular mechanism by which exercise‐induced endogenous β‐HB interacts with the cGAS‐STING pathway to confer liver protection. It provides novel insights into the anti‐aging properties of β‐HB in the liver, explores its potential as a novel nutritional supplement, and lays the groundwork for developing therapeutic strategies to delay liver aging.

## 2 Materials and Methods

### 2.1 Animal

SPF‐grade male C57BL/6J mice were procured from Gempharmatech (Jiangsu Jicui Yaokang Biotechnology Co. Ltd., China). The animals were categorized into two age groups: 6‐week‐old mice, housed until 4 months of age, constituted the young control (YC) group, whereas 6‐month‐old mice were kept until 15 months of age and subsequently randomized into four subgroups: old control (OC), old exercise (OE), old intervention with placebo (OIPA), and old intervention with β‐HB (OIPB). The mice were kept at ambient temperature (23°C ± 2°C) with a humidity of 50% ± 5%, a 12:12 h light–dark cycle, and unrestricted access to potable water and normal laboratory diet. Experimental interventions began at 15 months of age for the older cohorts, whereas the YC group received concurrent analyses to establish baseline comparisons. All animal research received approval from the Animal Care and Use Committee of Shanghai University of Sport (Ethics no: 102772023DW022).

### 2.2 Exercise‐Induced Experiment

The treadmill exercise program consists of 1 week of familiarization, followed by a 16‐week moderate‐intensity aerobic regimen, comprising 5 days per week of 1 h of continuous treadmill running at 15 m/min, with no slope (Donato et al. 2005). Throughout the treadmill training, we continuously monitored to ensure that all mice were running and not resting on the treadmill. This would ensure that all mice underwent an equivalent level of exercise training. The YC and OC groups remained inactive under the same housing conditions.

### 2.3 Exogenous β‐HB Administration

The OIPB group got intraperitoneal injections of β‐HB (200 mg/kg body weight), while the OIPA group was administered an equivalent volume of saline (Tan et al. 2022). Injections were given for 5 days per week for a duration of 16 weeks. β‐HB (Catalog no. H6501; Sigma, USA) was solubilized and diluted in sterile saline prior to application.

### 2.4 Measurement of β‐HB Levels in Blood

To evaluate the alterations in blood β‐HB levels induced by exercise, mice underwent a 1‐h treadmill session at a speed of 15 m/min. Blood was obtained from the tail vein before injection and at intervals of 15 min, 30 min, 1, 3, and 24 h post‐exercise, and the concentration of β‐HB was assessed using an Abbott ketone meter (FreeStyle Optium, YZB/UK 5991‐2012; Abbott, UK) (Roberts et al. 2017).

### 2.5 Cell Culture

AML12 mouse hepatocyte cells were cultured in DMEM/F‐12 with high glucose, 10% FBS, insulin‐transferrin‐selenium, 0.1 μM dexamethasone, and 1% penicillin–streptomycin. Raw264.7, mouse leukemia monocyte macrophage cells, were cultured in DMEM containing high glucose, 10% FBS, and 1% penicillin–streptomycin. AML12 and Raw264.7 were cultivated under usual conditions of 37°C, 95% air, and 5% CO_2_. A 1:3 ratio was employed for cell passage, utilizing cells in the exponential development phase for experimentation.

### 2.6 Cell Treatment Protocol

To develop a Dgal‐induced cellular aging model, cells were exposed to Dgal at several concentrations (50, 75, 100, 150, and 200 mM) for 24 h, and cytotoxicity was evaluated to identify the best concentration. β‐HB treatments (2.5, 5, 10, and 20 mM) were administered to assess dose–response effects and determine the best protective concentration. The experimental design for evaluating the STING signaling pathway contained the following conditions: control (Con), Dgal (100 mM, Dgal), Dgal + β‐HB (100 mM + 5 mM, DgalBHB), Dgal + DMXAA (150 μM, STING agonist), Dgal + β‐HB + c‐176 (1 μM, STING antagonist), and Dgal + β‐HB + DMXAA, utilizing Raw264.7 cells. Dgal and BHB were concurrently introduced into the cell culture medium at the specified concentration. DMXAA or c‐176 should be included in the culture medium 1 h prior, if required. Cells underwent treatment for 24 h prior to subsequent examination.

### 2.7 Conditioned Medium Collection and Treatment Assay

After the culture of RAW264.7 in standard medium (Con), Dgal (100 mM, Dgal), and Dgal + BHB (100 mM + 5 mM, DgalBHB) for 24 h, the cell supernatants were collected and centrifuged at 500 *g* for 10 min at a low temperature of 4°C. The conditioned medium (CM) was acquired following filtration through a 0.22 μm pore filter. Raw264.7‐CM was combined with double the volume of standard complete medium of AML12 to produce CM‐Con, CM‐Dgal, and CM‐DgalBHB, which were subsequently utilized to culture AML12 cells for 24 h.

### 2.8 ELISA

Serum parameters were assessed with ELISA kits in accordance with the manufacturer's guidelines. The Alanine aminotransferase (ALT) Test Kit (C009‐2‐1), Aspartate aminotransferase (AST) Test Kit (C010‐2‐1), Triglyceride (TG) Assay Kit (A110‐1‐1), Total Cholesterol (TC) Assay Kit (A111‐1‐1), Low‐density lipoprotein cholesterol (LDL‐C) determination reagent (A113‐1‐1), and High‐density lipoprotein cholesterol (HDL‐C) determination kit (A112‐1‐1) were acquired from JianCheng Bioengineering Institute.

### 2.9 H&E Staining, Immunofluorescence

The tissues were fixed in 4% paraformaldehyde and sectioned (5 μm) after being paraffin embedded. H&E staining and immunofluorescence were performed as described previously (Olympus microscope) (Zheng et al. 2023). For immunofluorescence, the cells were initially fixed in 4% paraformaldehyde for 10 min, thereafter rinsed three times with PBS, and then permeabilized with 0.2% Triton X‐100 for 5 min. The cells were washed with PBS, blocked with 10% BAS/PBS for 1 h, then incubated with primary antibodies at 4°C overnight. After three rinses with PBS, secondary antibodies were applied at room temperature for 1 h. The cells were subsequently rinsed thrice with PBS and mounted utilizing an anti‐fade mounting solution containing DAPI. The immunofluorescence‐stained cells were examined using a Leica microscope, and fluorescence intensity was assessed with ImageJ software.

### 2.10 Oil Red O Staining

Fresh liver tissue was wrapped in optimal cutting temperature compound, frozen, and sectioned (8 μm). Oil Red O staining was performed as described previously (Olympus microscope) (Wang et al. 2022). AML12 cells were subjected to three washes with cold PBS and subsequently fixed for 20 min using 4% formaldehyde. Oil Red O (Sigma; 0.5% in isopropanol) was diluted with water in a 3:2 ratio, filtered through a 0.45‐μm filter, and incubated with the fixed cells for 30 min at room temperature. The cells were rinsed with water, and the stained lipid droplets within the cells were analyzed using light microscopy and captured in photographs.

### 2.11 RNA Extraction and RT–qPCR Analysis

According to the manufacturer's instructions, total RNA was extracted from liver tissues (20–30 mg) or cell samples utilizing Trizol reagent (Invitrogen Life Technologies, Carlsbad, CA, USA), followed by quantification of the RNA concentration using a Nanodrop 2000 spectrophotometer (Thermo Fisher Scientific, San Jose, CA, USA). RNA was reverse transcribed with a RevertAid First Strand cDNA Synthesis Kit (Thermo Fisher Scientific) to synthesize cDNA for qPCR using SYBR Green PCR Master Mix (Vazyme Biotech Co. Ltd., Nanjing, China) in StepOnePlus (Applied Biosystems, Carlsbad, CA, USA). The mRNA expression content of target genes was standardized to the expression of β‐actin or 18 s, and was calculated with the $2^{-∆∆C_{T}}$ method. The mitochondrial RNA and cytosolic RNA were isolated by Tissue Mitochondria Isolation Kit (Beyotime, C3606).

### 2.12 Protein Extraction and Western Blot Analysis

Tissues and cells were harvested, prepared, and Western blotting was performed as described previously (Guo et al. 2024). The quantification of western blotting results was performed utilizing ImageJ. The BCA assay was employed for protein quantification. Proteins were denatured by combining them with 5× loading buffer. Western blotting was conducted by separating proteins at 90 V for 30 min, followed by 120 V for 1 h, then transferring them onto PVDF membranes at 110 V for 1.5 h. Incubation of primary and secondary antibodies was conducted on membranes. Protein signals were detected using chemiluminescence, and the images were processed with ImageJ software.

### 2.13 Cell Viability Assay

Cells were allocated into 96‐well plates at an approximate concentration of 1 × 10^4^ cells per well, with each well supplemented with 100 μL of complete media. Following a 24‐h incubation at 37°C in 5% CO_2_, the growth medium was replaced with either fresh complete medium or medium augmented with test chemicals at specified quantities. The cells were incubated for an additional 24 h, after which the medium was replaced with 100 μL of CCK‐8 solution diluted to one‐tenth of its original concentration. Thereafter, the plate underwent a 2‐h incubation, and the optical density at 450 nm was measured using a spectrophotometer.

### 2.14 Senescence‐Associated β‐Galactosidase Staining

Cells were allocated into 6‐well plates at an approximate concentration of 2 × 10^5^ cells per well and stained with the senescence β‐galactosidase (SA‐β‐Gal) Staining Kit (Beyotime, C0602) in accordance with the manufacturer's guidelines.

### 2.15 Identification of Free mtDNA in the Cytosol of Cells

To detect the level of free mtDNA in the cytosol of Raw264.7 cells, cells were collected following experimental treatments. Mitochondria were isolated using the Cell Mitochondria Isolation Kit (Beyotime, C3601) to obtain the cytosolic fraction. Subsequently, gDNA was extracted from the cytosolic fraction using the TIANamp Genomic DNA Kit (TIANGEN, DP304). Finally, the DNA levels of the mitochondrial gene *Nd1* and the nuclear gene *Cftr* were determined by quantitative PCR (qPCR). The relative expression level of *Nd1* was calculated using the Δ*C*_t_ method with *Cftr* as the reference gene to assess the enrichment of mtDNA in the cytosol.

### 2.16 Annexin V‐FITC/PI Apoptosis Assay

To assess the apoptosis of AML12 cells, the cells were stained utilizing the Annexin V‐FITC Apoptosis Detection Kit (Beyotime, C1062) according to the manufacturer's guidelines (Zhao et al. 2020). Annexin V‐FITC, which emits green fluorescence, identified early apoptotic cells, while PI, exhibiting red fluorescence, indicated necrotic or late apoptotic cells with damaged membranes.

### 2.17 Analysis of Public Datasets

The publicly accessible microarray dataset GSE133815 was analyzed to assess STING (TMEM173) expression levels in human liver tissues. This dataset contains transcriptomic profiles from 11 young individuals (21–45 years) and 9 aged individuals (61–80 years). Raw data files were retrieved from the NCBI GEO repository (https://www.ncbi.nlm.nih.gov/geo/query/acc.cgi?acc=GSE133815) for comparative analysis of STING expression between age groups.

### 2.18 Statistical Analysis

Data analysis was carried out with SPSS 22.0 and visualized with GraphPad Prism 8.0.2. Independent sample *t*‐tests were employed for comparing two groups. To compare multiple groups, one‐way ANOVA was conducted, followed by Bonferroni corrections. All data are expressed as mean ± standard deviation (SD). Statistical significance was defined as a *p*‐value under 0.05.

## 3 Results

### 3.1 Aerobic Exercise Promotes Endogenous Ketogenesis and Ameliorates Hepatic Lipid Deposition, Inflammation, and PANoptosis in Age Mice

**FIGURE 1:**
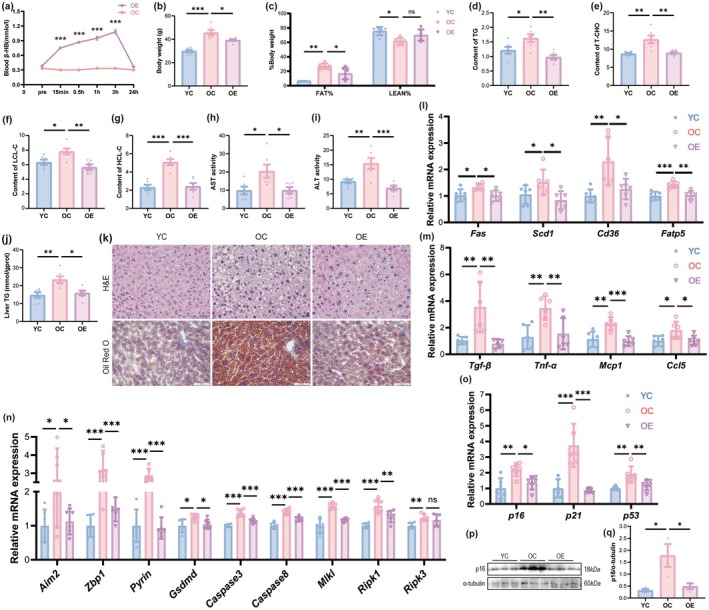
Aerobic exercise promotes endogenous ketogenesis and ameliorates hepatic lipid deposition, inflammation, and PANoptosis in the liver of aging mice. (a) Blood β‐HB levels in OC or OE groups at baseline and post‐exercise intervals (15 min, 30 min, 1, 3, and 24 h) (*n* = 6). (b) Body weight (*n* = 6). (c) Fat mass percentage (fat mass/body weight × 100%) and lean mass percentage (lean mass/body weight × 100%) (*n* = 6). (d–g) Serum levels of TC (d), TC (e), LCL‐C (f), and HCL‐C (g) (*n* = 6). (h, i) Serum activities of AST (h) and ALT (i) (*n* = 6). (j) Hepatic triglyceride (TG) content (*n* = 6). (k) Representative histological images of H&E staining (scale bar, 20 μm) and Oil Red O staining (scale bar, 50 μm) (l) The mRNA expression levels of lipid synthesis and transport genes (*n* = 6). (m) The mRNA expression levels of pro‐inflammatory cytokines (*n* = 6). (n) The mRNA expression levels of PANoptosis markers (*n* = 6). (o) The mRNA expression levels of senescence‐associated markers (*n* = 6). (p) Representative western blot images of p16. (q) Quantification of the protein expression level of p16 (*n* = 3). Data (mean ± SD) were analyzed using unpaired *t*‐tests (a) and one‐way ANOVA with Bonferroni post hoc tests (b–o). *p < 0.05, **p < 0.01, ***p < 0.001.

In order to investigate the dynamic changes in blood β‐HB levels induced by exercise, our study administered a single bout of exercise intervention (15 m/min, 1 h) to aged mice and measured their blood β‐HB levels before exercise and within 24 h post‐exercise. The results indicated that aerobic exercise substantially increased blood β‐HB levels, which returned to the baseline after 24 h (Figure 1a).

This study found that old mice, in contrast to young mice, demonstrated a large increase in body weight and body fat percentage, along with a substantial decrease in lean mass proportion. Aerobic exercise significantly reduced body weight growth and altered body composition in aged mice, notably by significantly reducing the rise in body fat percentage (Figure 1b,c). Subsequent examination of the four blood lipid parameters indicated that aerobic exercise markedly mitigated the heightened levels of serum TG, TC, LDL‐C, and HDL‐C in elderly mice (Figure 1d–g). Moreover, aerobic exercise substantially reduced the elevated blood activity of AST and ALT enzymes in aged mice, suggesting its ability to mitigate aging‐related liver damage (Figure 1h,i). To assess the impact of aerobic exercise on hepatic steatosis in aged mice, this study further measured hepatic TG levels and H&E staining and Oil Red O staining on liver tissues. The findings indicated that aerobic exercise markedly inhibited hepatic lipid deposition and the increase in TG levels in aged mice (Figure 1j,k). Examination of lipid metabolism‐associated genes demonstrated that aerobic exercise markedly decreased the hepatic mRNA expression levels of fatty acid synthesis proteins (*Scd1*, *Fas*) and fatty acid transport proteins (*Cd36*, *Fatp5*) (Figure 1l). Furthermore, aerobic exercise markedly reduced the hepatic mRNA expression levels of pro‐inflammatory cytokines (*Il‐1β*, *Tgf‐β*, *Mcp1*, *Ccl5*), PANoptosis‐related molecules (*Aim2*, *Zbp1*, *Pyrin*, *Gsdmd*, *Caspase3*, *Caspase8*, *Mlkl*, *Ripk1*, *Ripk3*), and senescence indicators (*p16*, *p21*, *p53*) in aged mice (Figure 1m–o). The protein level of p16 detected by Western blot was consistent with the qPCR results (Figure 1p,q). These results indicate that aerobic exercise collectively decreased body weight and body fat percentage in aged mice, enhanced hepatic lipid deposition, mitigated inflammatory responses and PANoptosis, consequently providing hepatoprotective effects.

### 3.2 Aerobic Exercise Suppresses Mitochondrial Dysfunction‐Associated cGAS‐STING Activation in Aged Mouse Liver

**FIGURE 2:**
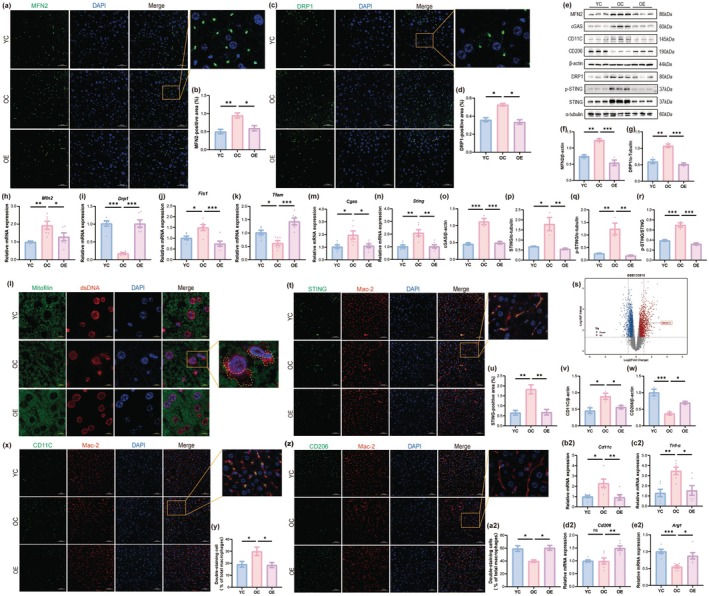
Aerobic exercise suppresses mitochondrial dysfunction‐associated cGAS‐STING activation in the liver of aged mice. (a) Representative images of MFN2 immunofluorescence staining (scale, 50 μm). (b) Quantification of MFN2‐positive area (*n* = 3). (c) Representative images of DRP1 immunofluorescence staining (scale, 50 μm). (d) Quantification of DRP1‐positive area (*n* = 3). (e) Representative western blot images of MFN2, DRP1, cGAS, p‐STING, STING, CD11C, and CD206. (f, g) Quantification of the protein expression level of MFN2 (f) and DRP1 (g) (*n* = 3). (h–k) mRNA expression levels of mitochondrial dynamics regulators: *Mfn2* (h), *Drp1* (i), *Fis1* (j), and *Tfam* (k) (*n* = 6). (l) Representative images of Mitofilin and dsDNA immunofluorescence staining (scale, 10 μm). (m, n) The mRNA expression levels of *Cgas* (m), *Sting* (n) (*n* = 6). (o–r) Quantification of the protein expression level of cGAS (o), STING (p), p‐STING (q), and p‐STING/STING (r) (*n* = 3). (s) Volcano plot of differentially expressed genes in elderly vs. young populations based on re‐analysis of GEO dataset GSE133815. (t) Representative images of STING immunofluorescence staining (scale, 50 μm). (u) Quantification of STING‐positive area (*n* = 3). (v, w) Quantification of the protein expression level of CD11C (v) and CD206 (w) (*n* = 3). (x) Representative images of CD11C and Mac‐2 immunofluorescence staining (scale, 50 μm). (y) Quantification of double‐staining area/Mac‐2‐positive area corresponding to (x) (*n* = 3). (z) Representative images of CD206 and Mac‐2 immunofluorescence staining (scale, 50 μm). (a2) Quantification of double‐staining area/Mac‐2‐positive area corresponding to (z) (*n* = 3). (b2–e2) The mRNA expression levels of macrophage polarization markers: *Cd11c* (M1), *Tnf‐α* (M1), *Cd206* (M2), and *Arg1* (M2) (*n* = 6). Data (mean ± SD) were analyzed using one‐way ANOVA with Bonferroni post hoc test. *p < 0.05, **p < 0.01, ***p < 0.001.

Mitochondrial dysfunction is a fundamental characteristic of aging. This work examined the role of mitochondrial homeostasis in the mechanism by which aerobic exercise reduces hepatic lipid accumulation in aged mice by evaluating the expression levels of markers associated with mitochondrial dynamics by immunofluorescence, qPCR, and Western blotting. Results from immunofluorescence (Figure 2a–d) and Western blotting (Figure 2e–g) indicated that aerobic exercise markedly reduced the protein expression levels of MFN2 and DRP1 in the livers of elderly mice. Aerobic exercise altered the mRNA expression levels of hepatic mitochondrial fusion protein (*Mfn2*), fission proteins (*Drp1*, *Fis1*), and biogenesis protein (*Tfam*), indicating that it can rectify aberrant mitochondrial dynamics in the livers of aged mice (Figure 2h–k).

Aberrant mitochondrial dynamics resulting in mtDNA release is a direct cause of various cellular damage. Cytosolic double‐stranded DNA (dsDNA) could originate from exogenous viral DNA, the release of nuclear DNA, or the release of mtDNA, with mtDNA being more readily released into the cytosol than nuclear dsDNA. This study employed double immunofluorescence staining to detect the expression and distribution of dsDNA in mouse livers. The findings indicated a substantial presence of free dsDNA in the cytoplasm of hepatocytes from elderly mice; aerobic exercise markedly diminished this aberrant release of dsDNA (Figure 2l).

The cGAS‐STING signaling pathway is recognized as the principal mechanism by which cells detect and respond to cytosolic free dsDNA. Our study found that both the gene (Figure 2m,n) and protein (Figure 2e,o,p) expression levels of cGAS and STING were significantly upregulated in the livers of aged mice, and more importantly, the levels of phosphorylated STING (p‐STING)—a direct indicator of pathway activation—and the p‐STING/STING ratio (Figure 2q,r) were also significantly increased. This confirms the activation of the pathway during aging. Notably, aerobic exercise effectively suppressed the overactivation of the cGAS‐STING signaling pathway, including significantly reducing p‐STING levels and its ratio. To investigate the expression changes of STING (also known as TMEM173) in human liver aging, we analyzed a gene expression profile database (GSE133815) comprising liver samples from 11 young controls and 9 aged individuals. The results demonstrated that STING expression in the liver was significantly upregulated in the aged population compared to the young controls (Figure 2s). This suggests that STING upregulation represents a conserved molecular alteration during liver aging. In liver tissue, STING is primarily expressed in macrophages. Immunofluorescence results indicated that STING colocalized with the macrophage marker Mac‐2, and its expression level was similar to the Western blot results (Figure 2t,u).

STING is closely associated with macrophage polarization, a process that significantly contributes to chronic inflammation during aging. To investigate the regulatory effect of aerobic exercise on hepatic macrophage polarization in aged mice, our study examined the regulatory influence of aerobic exercise on hepatic macrophage polarization in aged mice by measuring the protein expression levels of CD11C and CD206 through Western blotting (Figure 2e,v,w) and immunofluorescence (Figure 2x–a2), as well as evaluating the mRNA expression levels of M1 macrophage markers (*Cd11c*, *Tnf‐α*) and M2 macrophage markers (*Cd206*, *Cd163*) using qPCR (Figure 2b2–e2). The results indicated that the gene and protein expression levels of M1 markers were upregulated, while those of M2 markers were downregulated in the livers of aged mice; aerobic exercise significantly inhibited the polarization of hepatic macrophages towards the M1 pro‐inflammatory phenotype in aged mice.

In summary, in the livers of aged mice, a disruption in mitochondrial dynamics equilibrium results in elevated mtDNA release. The increased cytosolic free dsDNA induces the cGAS‐STING signaling pathway, promoting macrophage polarization towards the M1 pro‐inflammatory phenotype. Aerobic exercise can restore mitochondrial dynamics balance, inhibit cGAS‐STING pathway activation, and reverse the pro‐inflammatory phenotypic transformation of macrophages.

### 3.3 β‐HB Fails to Protect AML12 Hepatocytes Against Dgal‐Induced Senescence

**FIGURE 3:**
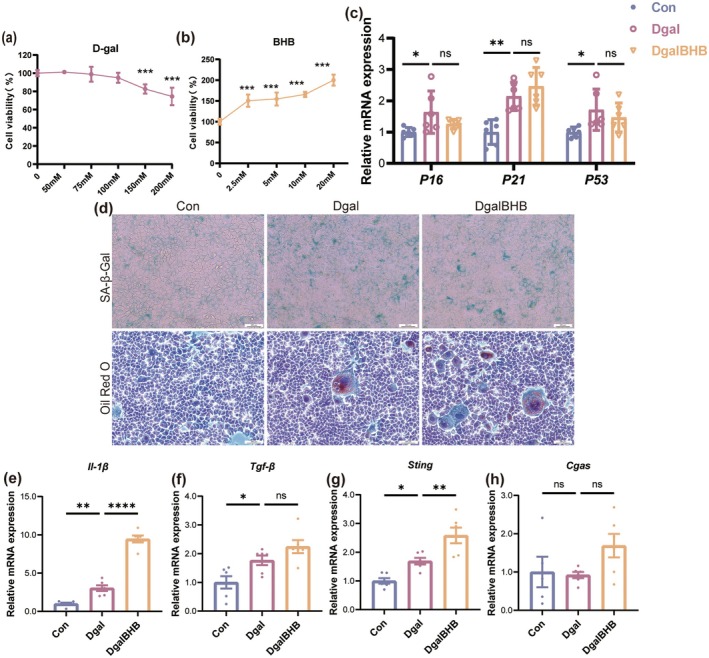
β‐HB fails to confer protection against Dgal‐induced senescence in AML12 hepatocytes. (a, b) CCK‐8 assay measuring cell viability after treatment with Dgal (a) or β‐HB (b) in AML12 cells (*n* = 6). (c) The mRNA expression levels of senescence‐associated markers in AML12 cells (*n* = 6). (d) Representative histological images of SA‐β‐Gal staining and Oil Red O staining (scale bar, 200 μm). (e–h) The mRNA expression levels of *Il‐1β* (e), *Tgf‐β* (f), *Cgas* (g) and *Sting* (h) in AML12 cells (*n* = 6). Data (mean ± SD) were analyzed using one‐way ANOVA with Bonferroni post hoc test. *p < 0.05, **p < 0.01, ***p < 0.001.

This work examined whether the exercise‐induced increase of β‐HB exerts its protective benefits on the aging liver by evaluating the direct impact of β‐HB on Dgal‐induced damage in AML12 mice hepatocytes. Initially, AML12 cells were subjected to several amounts of Dgal (50, 75, 100, 150, and 200 mM) or β‐HB (2.5, 5, 10, and 20 mM). Cytotoxicity assays indicated that elevated doses of Dgal were detrimental to AML12 cells (Figure 3a); therefore, 100 mM Dgal was chosen for subsequent treatments. Conversely, β‐HB at concentrations ranging from 2.5 mM to 20 mM exhibited no cytotoxicity (Figure 3b) and even enhanced cell viability; consequently, 5 mM β‐HB was chosen for the experiments. qPCR analysis demonstrated that treatment with 100 mM Dgal markedly increased the mRNA expression levels of senescence markers *p16*, *p21*, and *p53* in AML12 cells (Figure 3c). SA‐β‐gal staining and Oil Red O staining further validated that Dgal effectively triggered cellular senescence and lipid deposition in AML12 cells (Figure 3d). Furthermore, Dgal treatment elevated the mRNA expression levels of pro‐inflammatory cytokines *Il‐1β* and *Tgf‐β*, as well as *Sting*, although no significant alteration was noted in *Cgas* mRNA levels (Figure 3e–h). However, co‐treatment with 5 mM β‐HB failed to reverse Dgal‐induced senescence, lipid deposition, or inflammatory responses in AML12 cells. Collectively, these findings indicate that the hepatoprotective effects of β‐HB may not be mediated through its direct action on hepatocytes.

### 3.4 β‐HB Attenuates Dgal‐Induced M1 Polarization and Inflammatory Response in Raw264.7 Macrophages

Numerous published studies show that macrophages, such as Kupffer cells and infiltrating macrophages, are the main source of STING expression and activation in the livers of humans and mice (Yang et al. 2024; Luo et al. 2018; Thomsen et al. 2016). Additionally, our investigation revealed that the majority of STING expression is found in macrophages throughout the entire liver tissue.

**FIGURE 4:**
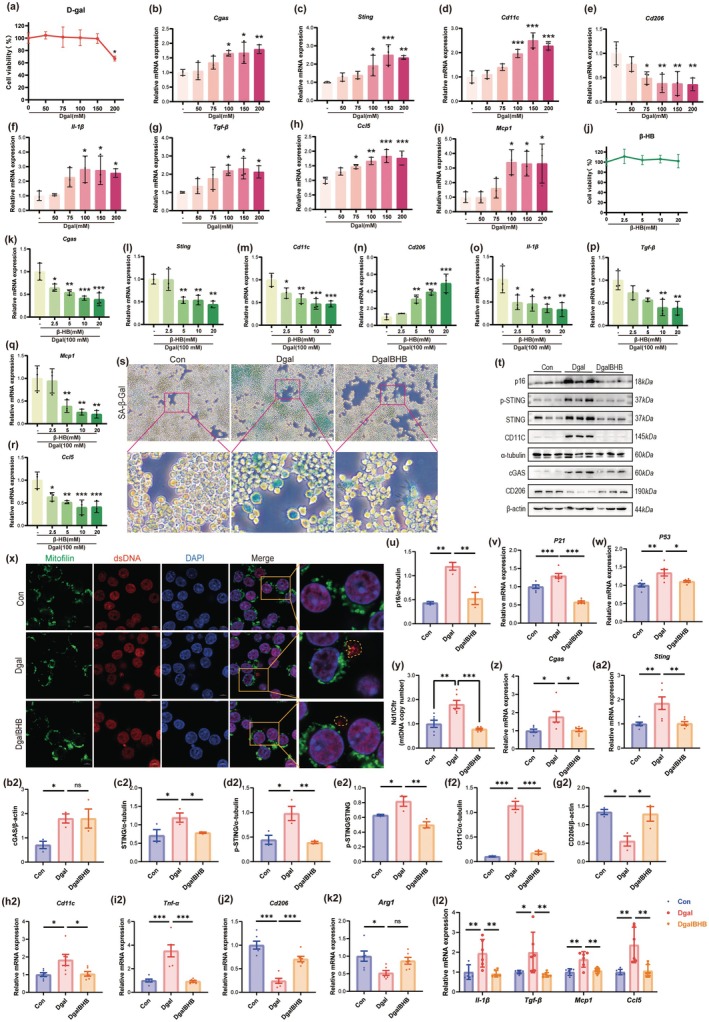
β‐HB attenuates Dgal‐induced M1 polarization and inflammatory response in Raw264.7 macrophages. (a) CCK‐8 assay measuring cell viability after treatment with Dgal in Raw264.7 cells (*n* = 6). (b–i) The mRNA expression levels of *Cgas* (b), *Sting* (c), *Cd11c* (d), *Cd206* (e), *Il‐1β*(f), *Tgf‐β*(g), *Ccl5*(h), and *Mcp1*(i) in Raw264.7 cells exposed to different concentrations of Dgal (0, 50, 75, 100, 150, and 200 mM) (*n* = 3). (j) CCK‐8 assay measuring cell viability after treatment with BHB in Raw264.7 cells (*n* = 6). (k–r) The mRNA expression levels of *Cgas* (k), *Sting* (l), *Cd11c* (m), *Cd206* (n), *Il‐1β* (o), *Tgf‐β* (p), *Ccl5* (q), and *Mcp1* (r) in Raw264.7 cells exposed to different concentrations of β‐HB (0, 2.5, 5, 10, and 20 mM) (*n* = 3). (s) Representative histological images of SA‐β‐Gal staining in Raw264.7 cells (scale bar, 200 μm). (t) Representative western blot images of p16, cGAS, STING, p‐STING, CD11C, and CD206. (u) Quantification of the protein expression level of p16 (*n* = 3). (v, w) The mRNA expression levels of *P21*, *P53* in Raw264.7 cells (*n* = 6). (x) Representative images of Mitofilin and dsDNA immunofluorescence staining in Raw264.7 cells (scale, 200 μm). (y) Free mtDNA levels in Raw264.7 cell cytoplasm (mitochondria‐depleted fraction) quantified by *Nd1/Cftr* ratio (*n* = 6). (z, a2) The mRNA expression levels of *Cgas* (z), *Sting* (a2) in Raw264.7 cells (*n* = 6). (b2–g2) Quantification of the protein expression level of cGAS (b2), STING (c2), p‐STING (d2), p‐STING/STING (e2), CD11C (f2), and CD206 (g2) (*n* = 3). (h2–k2) The mRNA expression levels of macrophage polarization markers: *Cd11c* (M1), *Tnf‐α* (M1), *Cd206* (M2), and *Arg1* (M2) (*n* = 6). (l2) The mRNA expression levels of pro‐inflammatory cytokines in Raw264.7 cells (*n* = 6). Data (mean ± SD) were analyzed using one‐way ANOVA with Bonferroni post hoc test. *p < 0.05, **p < 0.01, ***p < 0.001.

This investigation aims to ascertain if macrophage senescence correlates with STING pathway activation, M1 polarization, and inflammation. First, Raw264.7 cells were treated with different concentrations of Dgal (50, 75, 100, 150, and 200 mM) to establish a senescence model. CCK‐8 assays confirmed that Dgal doses of 20 mM or below were non‐cytotoxic (Figure 4a). qPCR analysis demonstrated that the mRNA expression levels of *Cgas* and *Sting* escalated with increasing Dgal concentrations (Figure 4b,c). Simultaneously, the mRNA expression of the M1 marker *Cd11c* escalated with concentration, but that of the M2 marker *Cd206* diminished (Figure 4d,e). Dgal administration elicited a dose‐dependent elevation of mRNA expression for several pro‐inflammatory cytokines (*Il‐1β*, *Tgf‐β*, *Mcp1*, and *Ccl5*) (Figure 4f–i). These results indicate that Dgal‐induced macrophage senescence correlates with the increase of the cGAS‐STING signaling pathway, M1 polarization, and inflammatory activity.

Subsequently, to investigate the effect of β‐HB, Raw264.7 cells were exposed to various concentrations of β‐HB (2.5, 5, 10, and 20 mM). CCK‐8 assays indicated that β‐HB concentrations ranging from 2.5 to 20 mM had no cytotoxic effect (Figure 4j). Under the influence of 100 mM Dgal stimulation, β‐HB administration substantially and dose‐dependently reduced the mRNA expression of *Cgas* and *Sting* (Figure 4k,l). Simultaneously, β‐HB downregulated the mRNA level of the M1 marker *Cd11c* and upregulated that of the M2 marker *Cd206* (Figure 4m,n), suggesting that β‐HB can inhibit the cGAS‐STING signaling pathway and reverse M1 polarization. β‐HB also dose‐dependently reduced the mRNA expression levels of pro‐inflammatory cytokines (Figure 4o–r), further validating its anti‐inflammatory effect.

To investigate the potential protective effects of β‐HB on Raw264.7 cells against Dgal‐induced pathological alterations, the cells were categorized into three groups: Control (Con), Dgal‐treated (Dgal, 100 mM), and Dgal plus β‐HB‐treated (DgalBHB, 100 mM Dgal + 5 mM β‐HB). The positive rate of SA‐β‐gal staining (Figure 4s), the protein level of p16 (Figure 4t,u), and the mRNA expression levels of senescence markers (*p21*, *p53*) (Figure 4v,w) indicated that β‐HB markedly suppressed Dgal‐induced cellular senescence. The immunofluorescence results indicated that β‐HB greatly inhibited Dgal‐induced cytoplasmic dsDNA levels (Figure 4x). mRNA levels and ratios of the mtDNA marker *Nd1* and the nuclear DNA marker *Cftr* in the cytoplasm (post mitochondrial extraction) were quantified by qPCR (Figure 4y). β‐HB mostly blocked the release of mtDNA into the cytoplasm.

The qPCR (Figure 4z,a2) and Western Blot (Figure 4z,b2–e2) results confirmed that β‐HB significantly inhibited the Dgal‐induced upregulation of the cGAS‐STING signaling pathway at both the mRNA and total protein levels, as well as the level of p‐STING. In addition, β‐HB inhibited the expression of M1 polarization markers while promoting the expression of M2 polarization markers (Figure 4f2–k2). Furthermore, β‐HB markedly suppressed Dgal‐induced mRNA expression of pro‐inflammatory cytokines (Figure 4l2), again confirming its anti‐inflammatory effect.

### 3.5 β‐HB Mediates Anti‐Senescence Effects in Raw264.7 Macrophages via STING Inhibition

**FIGURE 5:**
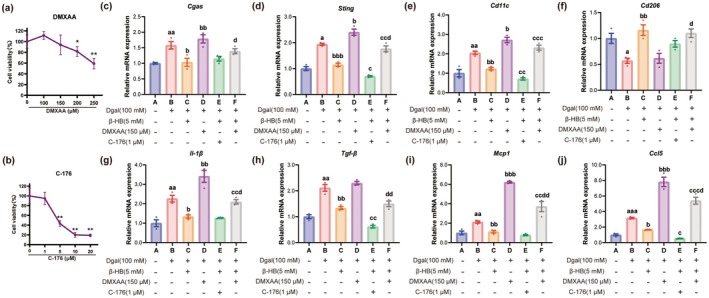
β‐HB mediates anti‐Senescence effects in Raw264.7 macrophages through STING inhibition. (a, b) CCK‐8 assay measuring cell viability after treatment with DMXAA (0,100, 200, and 250 μΜ, STING agonist) or C‐176 (0, 1, 5, 10, and 20 μΜ, STING antagonist) in Raw264.7 cells (*n* = 6). (c–j) The mRNA expression levels of *Cgas* (c), *Sting* (d), *Cd11c* (e), *Cd206* (f), *Il‐1β* (g), *Tgf‐β* (h), *Mcp1* (i), and *Ccl5* (j), in Raw264.7 cells (*n* = 3). Data (mean ± SD) were analyzed using one‐way ANOVA with Bonferroni post hoc test. *p* < 0.05 (a–d), *p* < 0.01 (aa–dd), and *p* < 0.001 (aaa–ddd) for comparisons vs. A (CON), B (Dgal 100 mM), C (Dgal 100 mM + β‐HB 5 mM), and D (Dgal 100 mM + DMXAA 50 μM).

This study aimed to determine if the protective effect of β‐HB is mediated by the suppression of the STING signaling pathway, utilizing the STING‐specific agonist DMXAA or the inhibitor C‐176 in a Dgal‐induced senescence paradigm with Raw264.7 cells. First, non‐cytotoxic working concentrations of DMXAA (150 μM) and C‐176 (1 μM) for Raw264.7 cells were determined using the CCK‐8 assay and used in subsequent experiments (Figure 5a,b). qPCR results (Figure 5c–j) indicated that DMXAA treatment (*Group D* vs. *Group B*) significantly enhanced Dgal‐induced mRNA expression of *Cgas* and *Sting*, while also increasing the expression of M1 polarization markers and pro‐inflammatory cytokines. This suggests that STING‐mediated macrophage M1 polarization and inflammatory responses play a role in Dgal‐induced senescence in Raw264.7 cells. Moreover, STING activation by DMXAA diminished the protective effect of β‐HB (*Group F* vs. *Group C*), but β‐HB mitigated the adverse effects caused by DMXAA (*Group F* vs. *Group D*). The data indicate that β‐HB confers its protective effect through the inhibition of the STING pathway. Additionally, treatment with the STING inhibitor C‐176 (*Group E* vs. *Group C*) further suppressed the mRNA expression of the M1 marker Cd11c and the pro‐inflammatory cytokines *Tgf‐β* and *Ccl5*, replicating the inhibitory effects of β‐HB on M1 polarization and inflammation.

This section demonstrates that pharmacological modulation of STING pathway activity in Raw264.7 cells reveals that STING pathway activation is a crucial mechanism driving Dgal‐induced macrophage M1 polarization and inflammatory responses. The protective effect of β‐HB on Raw264.7 cells is achieved by inhibiting the STING pathway.

### 3.6 β‐HB Inhibits Macrophage Senescence‐Induced Hepatocyte PANoptosis

**FIGURE 6:**
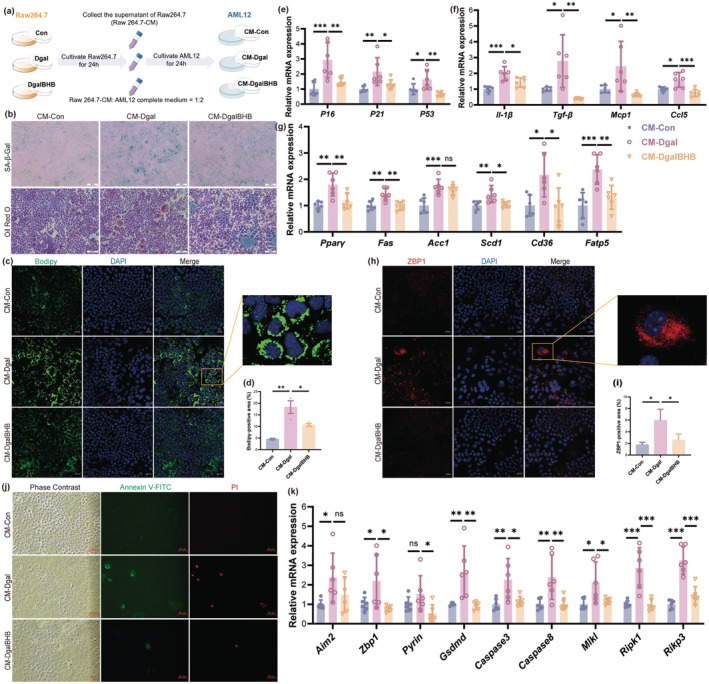
β‐HB inhibits macrophage senescence‐induced hepatocyte PANoptosis. (a) Schematic illustration of the experimental protocol: AML12 cells treated with conditioned medium from Raw264.7 cells. (b) Representative images of SA‐β‐Gal staining and Oil Red O staining in AML12 cell (scale, 200 μm). (c) Representative images of Bodipy immunofluorescence staining in Raw264.7 cells (scale, 20 μm). (d) Quantification of Bodipy‐positive area (*n* = 3). (e) The mRNA expression levels of senescence‐associated markers in AML12 cells (*n* = 6). (f) The mRNA expression levels of pro‐inflammatory cytokines in AML12 cells (*n* = 6). (g) The mRNA expression levels of lipid synthesis and transport genes in AML12 cells (*n* = 6). (h) Representative images of ZBP1 immunofluorescence staining in Raw264.7 cells (scale, 20 μm). (i) Quantification of ZBP1‐positive area (*n* = 3 per group). (j) Representative fluorescence images of AML12 cells showing PANoptosis markers, including Annexin V‐FITC (green), propidium iodide (PI, red), and bright‐field overlay (first three panels) (scale, 50 μm). (k) The mRNA expression levels of PANoptosis markers in AML12 cells (*n* = 6). Data (mean ± SD) were analyzed using one‐way ANOVA with Bonferroni post hoc test. *p < 0.05, **p < 0.01, ***p < 0.001.

Given that β‐HB supplementation did not directly reverse Dgal‐induced lipid deposition in AML12 hepatocytes, we hypothesized that β‐HB might indirectly regulate hepatocyte lipid deposition by protecting macrophages. To evaluate this idea, we collected CM from Raw264.7 subjected to three treatments: Con (Control), Dgal (100 mM), DgalBHB (100 mM + 5 mM). The CM was then mixed with twice its volume of complete AML12 hepatocyte medium and used to culture AML12 cells (corresponding groups: CM‐Con, CM‐Dgal, and CM‐DgalBHB) (Figure 6a). Results showed that CM from senescent Raw264.7 cells (CM‐Dgal) significantly increased the positive area of SA‐β‐GAL staining, Oil Red O staining, and Bodipy fluorescent staining (a lipid marker) in AML12 cells (Figure 6b–d). CM‐Dgal significantly increased the mRNA expression levels of senescence markers and pro‐inflammatory cytokines in AML12 cells, along with the mRNA levels of genes associated with lipid synthesis (*Pparγ*, *Fas*, *Acc1*, and *Scd1*) and lipid transport (*Cd36*, *Fatp5*) (Figure 6e–g). Nonetheless, the protective influence of β‐HB on senescent Raw264.7 cells (CM‐DgalBHB) markedly mitigated the adverse effects of CM on AML12 cells. In the mechanism exploration, the detection of the protein levels of the PANoptosis marker ZBP1 (Figure 6h,i), the Annexin V‐FITC/PI apoptosis assay assessing apoptosis and late necrosis (Figure 6j), and the qPCR analysis of mRNA levels of PANoptosis markers (Figure 6k) revealed that D‐galactose‐induced senescent Raw264.7 cells enhanced PANoptosis in AML12 cells. Conversely, β‐HB, by protecting macrophages from Dgal‐induced damage, significantly reduced the level of PANoptosis in hepatocytes.

In summary, β‐HB indirectly inhibited hepatocyte lipid deposition and PANoptosis by inhibiting the STING pathway in macrophages, attenuating macrophage M1 polarization and inflammatory responses.

### 3.7 Exogenous β‐HB Supplementation Mimics Aerobic Exercise‐Induced Hepatoprotective Effects of in Aged Mice

**FIGURE 7:**
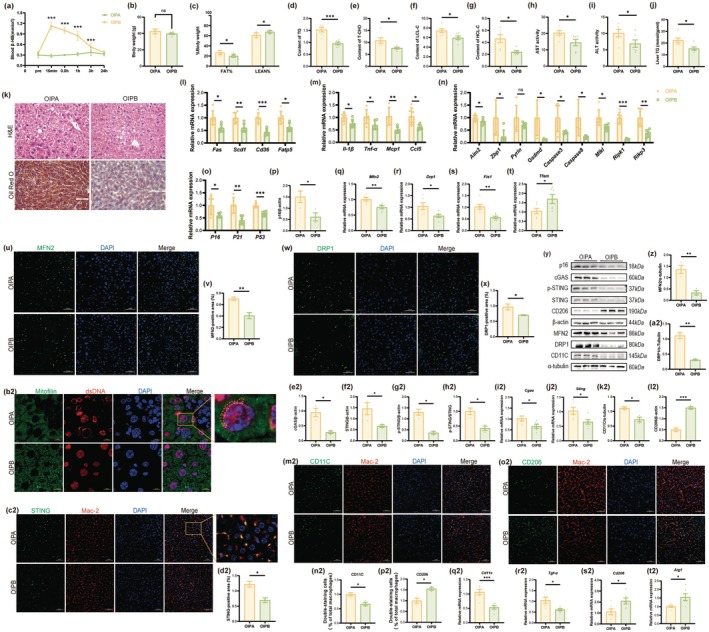
Exogenous β‐HB supplementation mimics the hepatoprotective effects of aerobic exercise in aged mice. (a) Blood β‐HB levels in the OIPA group or OIPB group at baseline and after a single β‐HB injection (15 min, 30 min, 1, 3, and 24 h) (*n* = 4). (b) Body weight (*n* = 6). (c) Fat mass percentage (fat mass/body weight × 100%) and lean mass percentage (lean mass/body weight × 100%) (*n* = 6). (d–g) Serum levels of TC (d), TC (e), LCL‐C (f), and HCL‐C (g) (*n* = 6). (h, i) Serum activities of AST (h), ALT (i) (*n* = 6). (j) Hepatic TG content (*n* = 6). (k) Representative histological images of H&E staining (scale bar, 20 μm) and Oil Red O staining (scale bar, 50 μm). (l) The mRNA expression levels of lipid synthesis and transport genes (*n* = 6). (m) The mRNA expression levels of pro‐inflammatory cytokines (*n* = 6). (n) The mRNA expression levels of PANoptosis markers (*n* = 6). (o) The mRNA expression levels of senescence‐associated markers (*n* = 6). (p) Quantification of the protein expression level of p16 (*n* = 3). (q–t) The mRNA expression levels of mitochondrial dynamics regulators: *Mfn2* (q), *Drp* (r), *Fis* (s), and *Tfam* (t) (*n* = 6). (u) Representative images of MFN2 immunofluorescence staining (scale, 50 μm). (v) Quantification of MFN2‐positive area (*n* = 3 mice per group). (w) Representative images of DRP1 immunofluorescence staining (scale, 50 μm). (x) Quantification of DRP1‐positive area (*n* = 3). (y) Representative western blot images of p16, MFN2, DRP1, cGAS, STING, p‐STING, CD11C, and CD206. (zz, a2) Quantification of the protein expression level of MFN2 (z) and DRP1 (a2) (*n* = 3). (b2) Representative images of Mitofilin and dsDNA immunofluorescence staining (scale, 10 μm). (c2) Representative images of STING immunofluorescence staining (scale, 50 μm). (d2) Quantification of STING‐positive area (*n* = 3). (e2–h2) Quantification of the protein expression level of cGAS (e2), STING (f2), p‐STING (g2) and p‐STING/STING (h2) (*n* = 3). (i2, j2) The mRNA expression levels of *Cgas* (i2), *Sting* (j2) (*n* = 6). (k2, l2) Quantification of the protein expression level of CD11C (k2) and CCD206 (l2) (*n* = 3). (m2) Representative images of CD11C and Mac‐2 immunofluorescence staining (scale, 50 μm). (n2) Quantification of double‐staining area/Mac‐2‐positive area corresponding to (m2) (*n* = 3). (o2) Representative images of CD206 and Mac‐2 immunofluorescence staining (scale, 50 μm). (p2) Quantification of double‐staining area/Mac‐2‐positive area corresponding to (o2) (*n* = 3). (q2–t2) The mRNA expression levels of macrophage polarization markers: *Cd11c* (M1), *Tnf‐α* (M1), *Cd206* (M2), and *Arg1* (M2) (*n* = 6). Data (mean ± SD) were analyzed using unpaired *t*‐test. *p < 0.05, **p < 0.01, ***p < 0.001.

To validate the role of β‐HB in vivo and investigate whether exogenous β‐HB could recapitulate the protective effects of exercise on the liver in aged mice, we administered β‐HB to aged mice via intraperitoneal injection (OIPB, 200 mg/kg BW). As expected, the exogenous β‐HB reached its peak more rapidly than exercise‐induced ketosis due to distinct pharmacokinetics. Nevertheless, both interventions produced a highly congruent blood ketone profile in terms of peak concentration and temporal duration (Figure 7a). Exogenous β‐HB supplementation had no significant effect on mouse body weight but significantly improved body composition, manifested as reduced fat mass and increased lean mass (Figure 7b,c). Moreover, β‐HB supplementation markedly reduced the levels of four serum lipid indicators and the enzymatic activities of liver damage markers (Figure 7d–i). Hepatic TG level detection and histological staining both showed (Figure 7j,k) that β‐HB supplementation significantly alleviated lipid deposition in the livers of aged mice, an effect closely associated with its regulation of the mRNA expression of lipid metabolism‐related proteins (Figure 7l). β‐HB supplementation also downregulated the mRNA expression levels of hepatic pro‐inflammatory cytokines, PANoptosis markers, and senescence markers (Figure 7m–o). Moreover, the attenuation of senescence was substantiated by a marked reduction in the protein level of p16 (Figure 7p,y). In summary, exogenous β‐HB supplementation had substantial protective effects on the livers of aged mice, principally evidenced by diminished hepatic lipid deposition, attenuated inflammatory responses, and reduced levels of PANoptosis. These effects were similar to those observed with exercise intervention.

In mechanistic exploration, detection of mitochondrial dynamics‐related parameters indicated that β‐HB supplementation significantly improved mitochondrial function (Figure 7q–a2). The mitochondrial protective effect diminished the release of cytosolic dsDNA (Figure 7b2), consequently reducing the overactivated cGAS‐STING pathway in the livers of aged mice (Figure 7y,e2–j2). Given that previous in vitro experiments confirmed that STING activation promotes macrophage M1 polarization, this study further evaluated the impact of β‐HB on macrophage polarization in the livers of aged mice. qPCR, Western blotting, and immunofluorescence data consistently indicated (Figure 7y,k2–t2) that exogenous β‐hydroxybutyrate supplementation inhibited the M1 polarization state and facilitated the transition towards an M2 phenotype in hepatic macrophages of aged mice.

## 4 Discussion

Although exercise is extensively documented to significantly delay liver aging and improve its function, the molecular mechanisms linking exercise stimuli to hepatic protective phenotypes remain unclear. Deciphering the exercise‐induced protective molecular mechanisms represents a key step in elucidating the mechanistic basis of exercise benefits (Chow et al. 2022). This study reveals that β‐HB serves as the central mediator underpinning the hepatoprotective effects conferred by aerobic exercise. Specifically, β‐HB mediates the protective effects of aerobic exercise on the aging murine liver by inhibiting the cGAS‐STING signaling pathway, resulting in diminished lipid deposition, reduced inflammatory responses, and decreased PANoptosis levels.

Throughout liver aging, diverse liver cells exhibit distinct phenotypic alterations. Senescent hepatocytes demonstrate enhanced lipid synthesis and uptake capacity, leading to hepatic lipid deposition, which correlates with altered transcriptional profiles of genes associated with steatosis (Seo et al. 2019; Nikopoulou et al. 2023). This research observed significant hepatic lipid deposition in naturally aged mice. Elevated expression levels of genes involved in lipid synthesis and uptake further indicate senescent hepatocytes exhibit lipid metabolism disorders. Notably, cellular senescence promotes hepatic lipid accumulation, while lipid deposition can stimulate hepatocyte senescence, establishing a vicious cycle (Lien et al. 2025). Research in high‐fat diet‐induced MASLD mouse models demonstrates that elevated hepatic triglyceride levels and lipid deposition promote increased expression of liver aging markers. Conversely, exercise can enhance hepatic lipophagy via activation of the AMPK/ULK1 pathway, reducing lipid deposition and postponing liver aging (Gao et al. 2020). Focusing on the exercise‐induced anti‐aging metabolite β‐HB, this research found that both exercise‐induced endogenous β‐HB generation and exogenous β‐HB supplementation significantly suppressed lipid deposition in the livers of aged mice. In validation experiments using the AML12 hepatocyte cell line, although Dgal effectively induced increased cellular senescence markers and lipid deposition, simultaneous treatment of β‐HB failed to mitigate Dgal‐induced damage in AML12 cells. Consequently, we determine that β‐HB does not impart its hepatoprotective benefits via directly modulating hepatocyte lipid metabolism or inhibiting hepatocyte senescence.

Macrophages, as pivotal regulators of hepatic immunity and inflammatory responses, are essential in regulating chronic inflammation associated with aging. Chronic inflammation hastens immune cell senescence and diminishes their capacity to eliminate senescent cells and cytokines, thereby intensifying inflammatory responses and creating a detrimental inflammation–senescence feedback loop. The anti‐inflammatory properties of β‐HB are well‐established (Han et al. 2020). This research further demonstrates that exercise‐mediated increases in β‐HB levels markedly reduce the inflammatory status in the livers of aged mice. We identified that the activation of hepatic inflammation in aged mice was mechanistically initiated by mitochondrial dysfunction, leading to the release of mtDNA into the cytosol. The cytosolic mtDNA activated the cGAS‐STING signaling pathway, leading to macrophage polarization towards the pro‐inflammatory M1 phenotype.

Mitochondrial dysfunction constitutes a fundamental characteristic of aging. The preservation of mitochondrial homeostasis depends on a set of interconnected mechanisms, including mitochondrial dynamics, mitochondrial biogenesis, and mitophagy. Altered mitochondrial dynamics have been shown to facilitate lipid accumulation in multiple tissues (e.g., liver, skeletal muscle, kidney) with age and significantly contribute to metabolic diseases such as obesity, metabolic syndrome, and T2DM (Zhao et al. 2014; López‐Lluch 2017; Meyer et al. 2017). Mitochondrial dynamics is a process whose state is impacted by various factors, including cell type, environmental stimuli, and genetic background. The related parameters display varying patterns across several illness models, indicating that targeting mitochondrial dynamics for specific diseases should focus on restoring equilibrium rather than merely augmenting or diminishing individual metrics (Wang et al. 2022). This research demonstrated that aerobic exercise markedly restored the disturbed equilibrium of mitochondrial dynamics in the livers of old mice. Mitochondrial transcription factor A (TFAM) is a crucial regulatory protein for mitochondrial biogenesis and is vital for sustaining mitochondrial quality control (Koh et al. 2021). Our results indicate that both aerobic exercise and exogenous β‐HB supplementation markedly restored the downregulation of TFAM mRNA expression levels in the livers of elderly mice. This finding aligns with previous research showing that high‐intensity interval training improves lipid metabolic disorders in T2DM mice by upregulating TFAM levels (Wang et al. 2022). Moreover, exogenous β‐HB supplementation efficiently replicated the endogenous ketogenic impact of aerobic exercise, reinstating mitochondrial homeostasis in the livers of elderly mice.

Mitochondrial homeostasis is essential for maintaining mtDNA copy number, integrity, and appropriate distribution. This study utilized Mitofilin/anti‐dsDNA dual immunofluorescence and observed a significant increase in free cytosolic dsDNA in hepatocytes of aged mice. Although this technique cannot definitively distinguish whether the cytoplasmic dsDNA originates from mitochondria or the nucleus, the combination of observed disordered mitochondrial dynamics and the altered ratio of Nd1 (a mtDNA gene) to Cftr (a nuclear gene) detected in the mitochondria‐depleted cytosol of Raw264.7 cells suggests that the increased cytoplasmic dsDNA is likely derived from mtDNA released by damaged mitochondria. Aerobic exercise and exogenous β‐HB supplementation both significantly reinstated mitochondrial homeostasis and decreased cytosolic free dsDNA. The finding was validated through cellular tests, in which β‐HB intervention effectively suppressed the Dgal‐induced increase in the Nd1/Cftr ratio in Raw264.7 cells. Cytosolic dsDNA functions as a damage‐associated molecular pattern (DAMP), activating DNA‐sensing pattern recognition receptors and initiating innate immune responses, a fundamental mechanism underlying aging‐related chronic inflammation (Newman and Shadel 2023). The cGAS‐STING signaling pathway serves as the central route for cellular sensing of cytosolic dsDNA threats, and its activation is induced by mtDNA release resulting from compromised mitochondrial homeostasis (Hu and Shu 2023; Kim et al. 2023). Analysis of the clinical dataset (GSE133815) demonstrated significantly upregulated STING expression in liver samples from aged individuals compared to young controls, showing high consistency with observations in murine models. This underscores the therapeutic potential of targeting STING signaling to mitigate liver aging. Our study provides translational evidence that aerobic exercise and exogenous β‐HB supplementation can indirectly suppress the aberrant activation of the cGAS‐STING pathway in aging livers by reinstating mitochondrial homeostasis, hence providing pragmatic options for fostering healthy aging.

The critical role of macrophages and macrophage‐mediated inflammatory responses in liver aging is well established (Hunt et al. 2019; Bloomer and Moyer 2021). Research demonstrates that STING can modulate macrophage polarization in diverse pathogenic conditions. Activation of STING in bone marrow‐derived macrophages promotes their polarization to the pro‐inflammatory M1 phenotype (Geng et al. 2023). Inhibition of the cGAS‐STING signaling pathway in macrophages promotes polarization towards the anti‐inflammatory M2 phenotype and reduces inflammation (He, Yue, et al. 2024; Li et al. 2024). The direct influence of liver aging on macrophage polarization is not fully comprehended. Recent studies indicate that aged macrophages display a propensity for pro‐inflammatory M1‐type polarization, potentially linked to the sustained activation of the cGAS‐STING pathway (Zhong et al. 2022). This study utilized immunofluorescence double‐staining for MAC‐2 and STING in liver tissue, revealing co‐localization of the STING protein with macrophages. Subsequent analysis of macrophage polarization markers indicated that liver macrophages in aged mice predominantly exhibited the M1 phenotype. Correspondingly, Western blotting and qPCR analyses of macrophage polarization and inflammation‐related markers demonstrated that β‐HB significantly inhibited the heightened M1 polarization in the livers of aged mice and facilitated a transition towards the M2 phenotype, thereby exerting anti‐inflammatory effects. The aforementioned data indicate that β‐HB could inhibit the STING pathway, thereby disrupting the detrimental cycle of macrophage polarization towards the pro‐inflammatory M1 phenotype in the aging microenvironment.

To validate in vitro whether the cGAS‐STING signaling pathway mediates M1 polarization in senescent macrophages, we first induced cellular senescence in Raw264.7 macrophage cells using Dgal. The results indicated that Dgal increased the mRNA levels of cGAS, STING, and the M1 marker CD11c in a dose‐dependent manner, while decreasing the mRNA level of the M2 marker CD206. This was accompanied by a simultaneous rise in the mRNA expression of pro‐inflammatory cytokines. Notably, co‐treatment with β‐HB markedly reduced cGAS and STING mRNA levels in a dose‐dependent manner and substantially mitigated Dgal‐induced M1 polarization and pro‐inflammatory responses in Raw264.7 cells. To further examine whether β‐HB's modulation of macrophage phenotypic switching and anti‐inflammatory actions is contingent upon the STING pathway, we altered STING expression in Dgal‐induced Raw264.7 cells utilizing the selective STING agonist DMXAA or the inhibitor C‐176. The results indicated that the STING agonist DMXAA intensified Dgal‐induced M1 polarization and inflammation in macrophages. More importantly, the protective effect of β‐HB on Raw264.7 cells was counteracted by STING activation. In contrast, the administration of the STING inhibitor C‐176 further decreased M1 polarization and inflammation, an outcome that aligns with the protective benefits of β‐HB. These results collectively indicate that β‐HB promotes the polarization of senescent macrophages towards the M2 phenotype and exhibits anti‐inflammatory effects by directly blocking the STING signaling pathway.

To clarify the indirect method through which β‐HB mitigates lipid accumulation in hepatocytes, we cultivated AML12 hepatocytes utilizing CM from Dgal‐treated Raw264.7 cells. The findings indicated that M1 polarization of macrophages and the inflammatory substances produced post‐Dgal induction considerably enhanced the expression of genes associated with lipid production and transport in AML12 cells, while also markedly elevating the expression of PANoptosis markers. Conversely, CM obtained from β‐HB‐pretreated Raw264.7 cells indirectly enhanced lipid accumulation and PANoptosis levels in AML12 cells.

PANoptosis is a unique inflammatory programmed cell death mechanism that encompasses the interactions of pyroptosis, apoptosis, and necroptosis. The PANoptosome complex mediates this process by integrating components from various cell death pathways (Sun et al. 2024). To date, Z‐DNA binding protein 1 (ZBP1)‐, Absent in Melanoma (AIM)‐, receptor‐interacting serine/threonine‐protein kinase 1 (RIPK1)‐, and NLR family pyrin domain containing 12 (NLRP12)‐ PANoptosomes have been characterized at the molecular level (Pandeya and Kanneganti 2024). PANoptosis is an important component of innate immune responses and is linked to various infections, inflammatory disorders, and malignancies. The function of PANoptosis in cellular senescence and age‐associated chronic inflammation remains unclear. p53, a crucial regulator of cell proliferation, senescence, DNA repair, and apoptosis, is frequently utilized as a biomarker for cellular senescence. The activation of this process might induce chronic inflammation and PANoptosis pathways, indicating that PANoptosis may facilitate the inflammatory response in cellular senescence (He, Tang, et al. 2024). The cGAS‐STING pathway, a critical sensor for dsDNA threats in the innate immune system, has been shown to trigger PANoptosis‐mediated inflammatory responses upon activation in multiple tissues including the liver, kidney, and lungs (Wu et al. 2025; Yi et al. 2024; He, Deng, et al. 2023). This study identified markedly increased levels of PANoptosis in the liver of aged mice and in hepatocytes, corroborating the role of PANoptosis in facilitating age‐related chronic inflammation. Aerobic exercise‐induced endogenous β‐HB synthesis and exogenous β‐HB supplementation markedly decreased PANoptosis levels. Mechanistically, β‐HB indirectly downregulated PANoptosis in hepatocytes by inhibiting the cGAS‐STING pathway in senescent macrophages. This study has elucidated the significance of the β‐HB/cGAS‐STING/PANoptosis axis in the aging liver; however, it has not yet thoroughly identified the components produced by M1‐type macrophages that specifically induce PANoptosis in senescent liver cells. Recent evidence indicates that IL‐1β is both a constituent of PANoptosis cell death and one of its effector output factors. Furthermore, an in vitro indirect co‐culture experiment demonstrated that Maresin, a mediator produced from macrophage docosahexaenoic acid, can reduce hepatocyte apoptosis by decreasing the levels of ROS, IL‐1β, and TNF‐α in macrophages. We hypothesize that in the aging liver microenvironment, M1‐type macrophages are highly likely to induce hepatocyte PANoptosis through the paracrine secretion of factors such as IL‐1β. However, this hypothesis demands additional experimental validation. This study is the first to report the mechanism by which exercise‐induced β‐HB regulates macrophage polarization by inhibiting the cGAS‐STING pathway, and thereby indirectly inhibiting hepatocyte PANoptosis. Although the specific effector molecules derived from macrophages remain incompletely elucidated, the upstream signaling pathway (cGAS‐STING) and the principal mediator (β‐HB) identified in this study offer a significant theoretical foundation for comprehending the molecular mechanisms of liver aging and formulating targeted intervention strategies.

This study provides significant insights into the β‐HB‐mediated pathways that safeguard against liver aging, but it also has several limitations that require further exploration.

Initially, while we have identified that exogenous supplementation of β‐HB can simulate some of the protective effects of exercise and have tentatively established that this effect relies on the inhibition of the macrophage cGAS‐STING pathway, we have not yet directly confirmed the causal relationship between exercise and β‐HB through genetic manipulation. Employing specific inhibition of the key ketogenic enzymes HMGCS2 or BDH1 in aged mice to suppress endogenous β‐HB production and subsequently evaluating whether the hepatoprotective effects of exercise are attenuated or whether exogenous β‐HB supplementation can restore these effects would provide more direct experimental evidence for the role of exercise‐induced β‐HB in mediating hepatic protection. Moreover, the inhibitory impact of β‐HB on the cGAS‐STING pathway has predominantly been confirmed at the cellular level. Future research should utilize animal models to selectively knock out or activate the STING signaling pathway in macrophages to investigate whether the protective effects of exercise or β‐HB persist.

Another limitation lies in the fact that thisresearch predominantly utilizes a naturally aged mouse model and a singular cell line system (Raw264.7 macrophages/AML12 hepatocytes), without confirmation in human primary hepatocytes or tissue specimens. While the Dgal‐induced cellular senescence model is widely used, the molecular characteristics of immortalized cell lines differ from natural aging states, which may limit the physiological relevance of the findings.

The rapid advancement of high‐throughput sequencing technologies has demonstrated the importance of single‐cell and spatial transcriptomics in elucidating cellular heterogeneity within tissue microenvironments, gene expression dynamics, and intercellular communication, thereby significantly enhancing our mechanistic comprehension of complex biological processes. This study initially indicates that β‐HB may mitigate liver aging by modulating macrophage‐hepatocyte interactions; however, we fully recognize that further single‐cell level analysis, particularly through the use of scRNA‐seq or snRNA‐seq on liver tissues from young, aged, exercise‐intervened, and β‐HB‐treated mice, could systematically uncover finer transcriptional landscapes across hepatocyte and immune cell subsets. Moreover, conducting bulk RNA‐seq on in vitro β‐HB‐treated hepatocytes and macrophages, followed by integrated comparison with in vivo transcriptional profiles, could provide systematic evidence regarding the mechanisms by which exercise and β‐HB counteract liver aging and may uncover novel regulatory targets and signaling networks.

This work validates that exercise elevates blood ketone levels and that exogenous β‐HB mimics its protective benefits; however, it is crucial to note that the metabolic responses triggered by exercise encompass much more than ketogenesis. A comprehensive non‐targeted metabolomic analysis of fluctuations in blood metabolites pre‐ and post‐exercise may elucidate the full extent of exercise‐induced metabolic remodeling, thereby revealing additional advantageous metabolites beyond β‐HB and enhancing understanding of the metabolic mechanisms driving the systemic anti‐aging effects associated with exercise. The integration of multi‐omics data will be a primary emphasis of future research, offering enhanced theoretical foundations and practical approaches for therapeutic dietary interventions and the prevention and treatment of age‐related hepatic disorders.

## 5 Conclusion

This study identifies β‐hydroxybutyrate (β‐HB) as an endogenous exercise mimetic that mediates the protective benefits of aerobic exercise against age‐related liver deterioration. We illustrate that exogenous β‐HB supplementation effectively replicates various exercise‐induced hepatoprotective effects, including enhanced mitochondrial function, reduction of chronic inflammation, and retarded PANoptosis, thereby providing a viable nutritional intervention strategy for individuals constrained by age or health conditions that impede physical activity. This study is the first to accurately identify the effect of β‐HB within hepatic macrophages, demonstrating that it inhibits the activation of the crucial innate immune signaling hub STING, thus promoting macrophage polarization towards the M2 anti‐inflammatory phenotype and indirectly improving hepatocyte function. This newly recognized intercellular communication mechanism offers a novel perspective on the metabolic–immune interactions linked to exercise. Our findings enhance the molecular comprehension of exercise effects and, crucially, offer direct theoretical and experimental validation for the development of β‐HB‐based anti‐aging therapies. Such strategies possess considerable clinical translational promise and may function as innovative alternative methods for enhancing liver health in the elderly, especially in individuals with limited exercise capacity.

## Author Contributions

**Ke Li** and **Weihua Xiao:** conceived and designed the study, and wrote the manuscript. **Lian Wang** and **Danlin Zhu:** performed animal husbandry/cultivation. **Ke Li** conducted the animal and cell experiments. **Yifan Guo** and **Wenhong Wang:** provided technical guidance. **Haoyang Gao** and **Muge Zhou:** participated in the interpretation of the results. **Ke Li** and **Weihua Xiao:** finalized the manuscript. All authors critically read and commented on the final manuscript.

## Funding

This work was sponsored by the National Natural Science Foundation of China (32371185), the Shanghai Science and Technology Plan Project (23010504200), the Key Lab of Exercise and Health Sciences of Ministry of Education (Shanghai University of Sport) (2025KF002), the Shanghai Key Lab of Human Performance (Shanghai University of Sport) (No. 11DZ2261100), and the Shanghai Oriental Talents Program (Youth Project). We thank BioRender for providing drawing support.

## Conflicts of Interest

The authors declare no conflicts of interest.

## Data Availability

The data that support the findings of this study are available on request from the corresponding author. The data are not publicly available due to privacy or ethical restrictions.
